# Eu_5_VO_10_: Synthesis Methods and Characterization of Basic Physicochemical Properties

**DOI:** 10.3390/ma19132782

**Published:** 2026-07-01

**Authors:** Kamil Kwiatkowski, Elżbieta Filipek, Mateusz Piz, Paweł Kochmański

**Affiliations:** 1 Department of Inorganic and Analytical Chemistry, Faculty of Chemical Technology and Engineering, West Pomeranian University of Technology in Szczecin, al. Piastów 42, 71-065 Szczecin, Poland; elzbieta.filipek@zut.edu.pl; 2 Faculty of Mechanical Engineering and Mechatronics, West Pomeranian University of Technology in Szczecin, al. Piastów 19, 70-310 Szczecin, Poland; pawel.kochmanski@zut.edu.pl

**Keywords:** Eu_5_VO_10_, high-temperature solid-state reaction, mechanochemical synthesis, modified Pechini method, XRD, UV-Vis-DRS, semiconductor

## Abstract

Rare-earth vanadates constitute an important class of functional materials with potential applications as luminophores, in optoelectronics and catalysis. The research for this work was inspired by the incomplete literature data, including the synthesis, structure and physicochemical properties of europium(III) vanadate(V) with the general formula Eu_5_VO_10_. The primary goal of this work was to supplement the missing data about this compound and identify its potential applications. This compound was synthesized using three methods, including waste-free methods: ceramic, mechanochemical and a modified Pechini method. The obtained Eu_5_VO_10_ was characterized using XRD, DTA–TG, FTIR, UV–Vis–DRS, SEM and gas pycnometry. It was settled that Eu_5_VO_10_ crystallizes in the monoclinic system and is thermally stable up to a temperature of approximately 1310 °C, above which it decomposes in the solid phase. Estimated energy gap (E_g_) values ranged from ~3.21 eV to ~3.53 eV depending on the synthesis method used, allowing Eu_5_VO_10_ to be classified as a wide-bandgap electrical semiconductor. The results also showed that the synthesis method affects the crystallite size of the synthesized compound. The development of synthesis methods and characterization of Eu_5_VO_10_ expands our understanding of rare-earth vanadates and their potential applications as functional materials.

## 1. Introduction

The results of basic research, particularly in the field of solid state chemistry and physicochemistry, play an important role in modern materials engineering, which is focused on the production of new materials using previously unknown phases, including chemical compounds, of key importance to the chemical, electronics and automotive industries.

Knowledge of synthesis methods and conditions, as well as the basic physicochemical properties of new phases, is essential for undertaking further applied research. For example, studies of binary and multicomponent systems of various compounds, including rare earth element (REE) oxides, enable researchers to gain insight into the solid phases formed in these systems. Determining their properties, such as thermal stability, energy gap value, and crystalline structure, is crucial for predicting the physical properties of materials with more complex compositions and, therefore, diverse applications. Knowledge of favourable conditions for the synthesis and phase transformations of new compounds forms the basis for rational management of raw materials, including REE compounds, in line with the concept of sustainable development by eliminating inefficient implementation steps.

Of particular interest from the application point of view are phases containing europium, including europium(III) orthovanadate(V), which, due to its luminescent and magnetic properties, can be used as a component of cathodoluminescent materials, thermoluminophores, scintillators, and crystal lasers [1,2,3,4,5,6,7,8,9]. Also, the presence of Eu^3+^ ions in the structures of orthovanadates(V) of other elements, e.g., in YVO_4_, due to their high thermal stability and high quantum efficiency, enables the use of such phases as components in measuring instruments, optoelectronic devices or biochemical markers [1,2,3,4,5,6,7,8,9]. Structural parameters such as crystallinity, particle size, morphology, and defect concentration significantly influence the optical, electrical, magnetic, and photocatalytic behaviour of these materials by modifying charge-carrier dynamics and radiative recombination processes [1,2,3,4,5,6,7,8,9]. Especially in terms of high thermal and chemical stability, REE orthovanadates(V) are meant to be important for many purposes, as they are better than sulfide-based phosphors [1,2,3,4,5,6,7,8,9]. Also, one of the most important and popular application of these phases are as different light sources, where due to the presence of REE in the structure, these f-block elements trivalent cations emit a wide range of wavelengths [1,2,3,4,5,6,7,8,9].

Despite intensive research on binary oxide systems V_2_O_5_–RE_2_O_3_, europium(III) vanadates(V) with stoichiometry other than europium(III) orthovanadate(V)—EuVO_4_ remain poorly characterized. The limited literature contains fragmentary and often divergent data [10,11,12]. Basic physicochemical properties of EuVO_4_, such as structure, optical, electrical, magnetic, and catalytic properties, as well as its applications are known [1,2,3,4,5]. On the other hand, phases with other europium contents, such as Eu_5_VO_10_, Eu_8_V_2_O_17_ or Eu_3_VO_7_, have only been mentioned in the literature [10,11,12].

Based on the available literature data, it has been established that the most frequently mentioned—apart from EuVO_4_—europium(III) vanadate(V) is the compound with the formula Eu_5_VO_10_, written also as 5Eu_2_O_3_·V_2_O_5_ or Eu_10_V_2_O_20_. However, the physicochemical characterization of this compound remains incomplete, including information about its structure, thermal stability, or phase transitions, which hinders its reproducible synthesis and prevents the indication of its potential applications. From the literature data, it is only known that the compound Eu_5_VO_10_ is formed by heating a mixture of Eu_2_O_3_ with V_2_O_5_ in a 5:1 molar ratio in 24 h steps in the temperature range of 600–1500 °C [10]. According to another work [11], the synthesis of this compound begins only at ~1200 °C.

The main goal of the presented research was to confirm the formation of the Eu_5_VO_10_ compound by high-temperature solid-state reactions and to determine whether this compound can also be obtained by other methods, i.e., a mechanochemical method and the modified Pechini method. Furthermore, the scope of this work included determining the final composition of this obtained compound, calculating its unit cell parameters, checking its thermal stability and estimating the energy band gap (E_g_).

The results of the conducted research included, among other things, confirming the formation of the Eu_5_VO_10_ phase using three selected methods. Furthermore, basic crystallographic data and its thermal stability were determined, and the obtained vanadate was classified as an electrical semiconductor. This further expands our knowledge of europium(III) vanadate(V) and opens up new possibilities for the engineering of functional materials based on this phase.

## 2. Materials and Methods

The following reagents were used to carry out the syntheses: europium(III) oxide (99.9%, Alfa Aesar, Karlsruhe, Germany), vanadium(V) oxide (99.9%, Alfa Aesar, Karlsruhe, Germany), ammonium metavanadate(V) (99.9%, POCh, Gliwice, Poland), aqueous ammonia solution (99.9%, Stanlab, Lublin, Poland), aqueous nitric acid solution (99.9%, Stanlab, Lublin, Poland), tartaric acid (99.9%, POCh, Gliwice, Poland), and glycerol (99%, POCh, Gliwice, Poland).

The synthesis of pentaeuropium decaoxovanadate (Eu_5_VO_10_) was carried out by three methods: high-temperature solid-state reactions (ceramic method), the mechanochemical method and the modified Pechini method.

**Figure 1:**
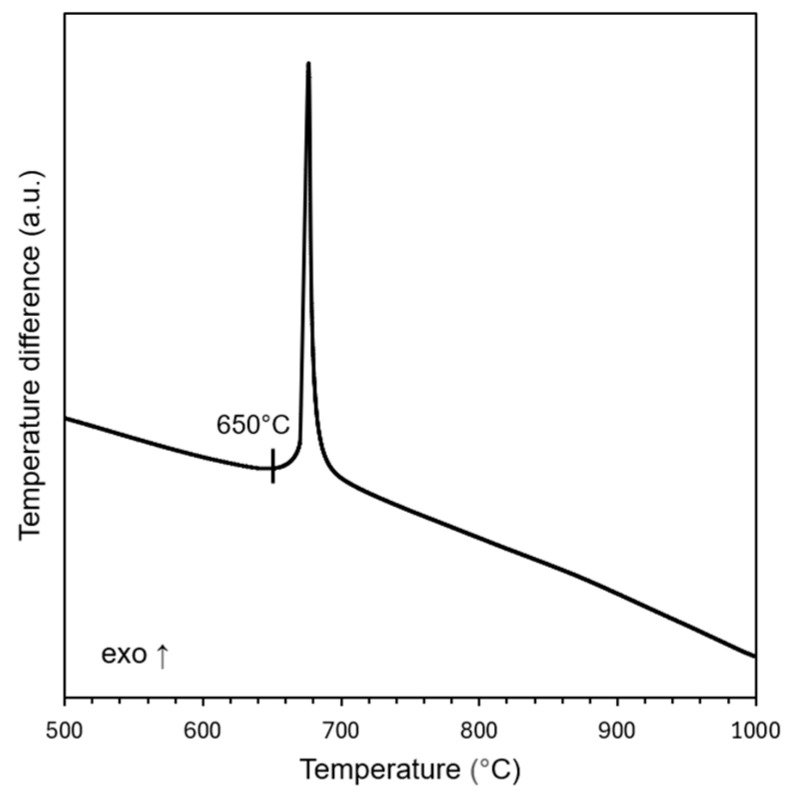
A fragment of the DTA curve of initial oxides mixture (V_2_O_5_:Eu_2_O_3_ molar ratio of 1:5).

**Figure 2:**
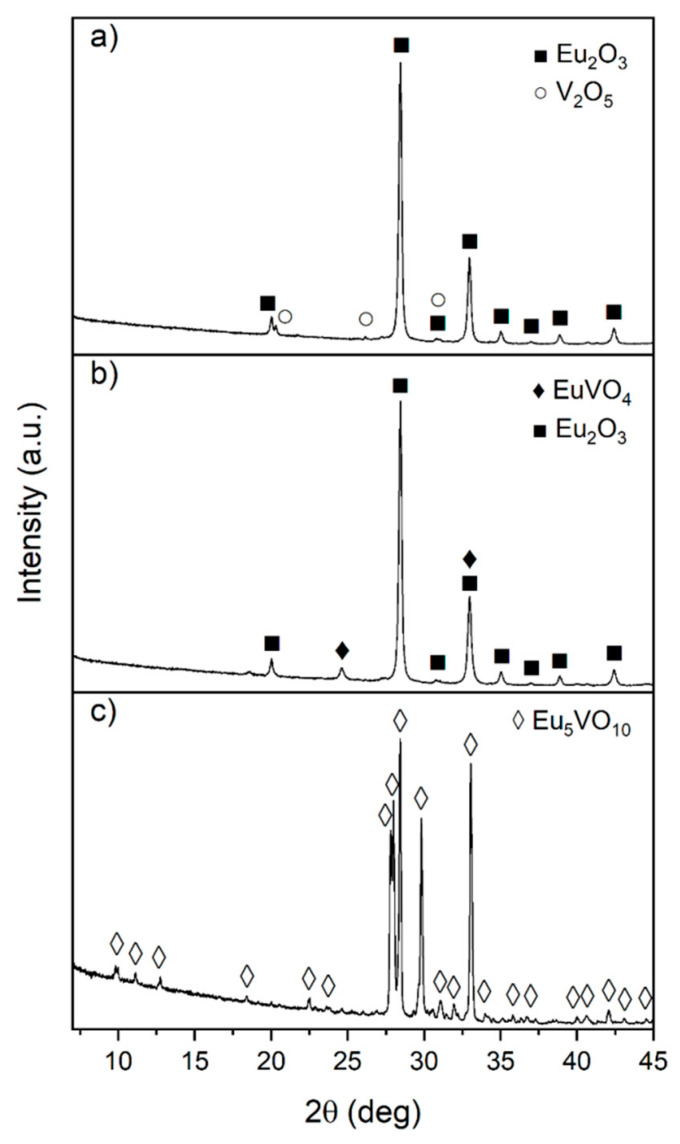
Fragments of the diffractograms: (**a**) mixture of substrates; (**b**) sample after the first synthesis step (600 °C, 12 h); (**c**) after the last synthesis step (1300 °C, 12 h).

To obtain Eu_5_VO_10_ by high-temperature solid-state reactions in the air atmosphere, a mixture of vanadium(V) oxide and europium(III) oxide was prepared in a V_2_O_5_:Eu_2_O_3_ molar ratio of 1:5. The homogenized oxide mixture was heated in five (I-V) 12 h stages in a muffle furnace (Carbolite, Hope Valley, UK) at temperatures: I: 600 °C; II: 630 °C; III: 1200 °C; IV: 1250 °C; V: 1300 °C. The temperature of the first stage of synthesis was determined based on DTA analysis of the substrate mixture, i.e., V_2_O_5_ and Eu_2_O_3_. The DTA curve registered from 20 to 1000 °C (Figure 1) at a temperature of approximately 650 ± 5 °C showed the onset of only one well-developed exothermic effect associated with the synthesis of EuVO_4_ (as confirmed by XRD phase analysis of the same sample after DTA analysis to 1000 °C). The registered diffractogram of this sample was similar to the one presented in Figure 2b. However, to avoid melting components of the reaction mixture (i.e., below the melting point of V_2_O_5_ (Tm = 675 °C) [13]) as well as based on the information about similar M_5_VO_10_ vanadate [13] it was settled that synthesis should be initiated at the temperature 600 °C.

The temperature of the second stage was increased, but only by 30 °C, which was selected to avoid melting the reaction mixture, especially leftover V_2_O_5_ in this sample.

To obtain Eu_5_VO_10_ by the second method, i.e., the mechanochemical (high-energy grinding) method, the prepared stoichiometric mixture of vanadium(V) oxide and europium(III) oxide was ground in a planetary ball mill in 15 min stages (a total of 10 h) in a Pulverisitte 6 mill (Fritsch, Idar-Oberstein, Germany). The reaction mixture was placed in a reactor (250 cm^3^, ZrO_2_) with grinding balls (20 mm in diameter, ZrO_2_), maintaining a BPR (ball to powder ratio) of 20:1 and a rotation speed of 620 RPM (revolutions per minute). The synthesis conditions were selected based on the literature data, available, among others, in [13,14].

For the synthesis of Eu_5_VO_10_ using the modified Pechini method, two solutions, A and B, were prepared. Solution A was prepared by dissolving europium(III) oxide in 6 mol/dm^3^ nitric acid, and then adding tartaric acid and glycerol in such an amount that the molar ratio of Eu(NO_3_)_3_:C_4_H_6_O_6_:C_3_H_5_(OH)_3_ was 3:1:3. Solution B was ammonium metavanadate(V) dissolved in 3 mol/dm^3^ aqueous ammonia solution. After 15 min of stirring solution A with a magnetic stirrer, solution B was added to the solution to maintain a Eu:V molar ratio of 5:1 in the reaction mixture. The resulting precipitate was heated, with constant stirring, at 50 °C until the solvent evaporated. The product was first dried at 100 °C for an hour and then calcined at 1300 °C (1 h). The conditions for synthesis using this method were determined based on the literature data regarding the preparation of various oxide phases [15,16,17].

During the conduction of this research, the following methods were used:

- X-ray powder diffraction (Empyrean II diffractometer (PANalytical, Almelo, The Netherlands) with copper lamp CuKα = 0.15418 nm);
- Differential thermal analysis with simultaneous thermogravimetry (Discovery SDT 650 thermal analyzer (TA Instruments, New Castle, DE, USA) in temperature range 20–1400 °C) and derivatograph F.Paulik, J.Paulik, L.Erdey (MOM, Budapest, Hungary), in temperature range 20–1000 °C;
- Fourier transformed infrared spectroscopy (Nicolet iS5 spectrometer (Thermo Fisher Scientific, Waltham, MA, USA) in wavenumber range 400–4000 cm^−1^);
- Ultraviolet and visible light diffuse reflectance spectroscopy (V-670 spectrophotometer (Jasco, Tokyo, Japan) in wavelength range 200–800 nm);
- Gas pycnometry (Ultrapyc 1200e ultrapycnometer (Quantachrome Instruments, Boynton Beach, FL, USA) in the 5N argon gas);
- Scanning electron microscopy with energy dispersive X-ray spectroscopy (SU-70 microscope (Hitachi, Tokyo, Japan) equipped in EDX NORAN System 7 spectrometer (Thermo Fisher Scientific, Waltham, MA, USA).

## 3. Results and Discussion

### 3.1. XRD

Based on qualitative phase analysis using XRD, it was confirmed that the Eu_5_VO_10_ compound was formed by stepwise heating of a mixture of oxides in a molar ratio of Eu_2_O_3_:V_2_O_5_ of 5:1. Figure 2 shows fragments of the diffraction patterns: the substrate mixture (a), this mixture after the first stage of its heating, i.e., at 600 °C (b), and the sample after the last stage of synthesis at 1300 °C (c).

In the registered diffraction pattern of the reaction mixture after the first stage of its heating (Figure 2b), apart from the diffraction lines belonging to the regular Eu_2_O_3_ set (PDF card no. 04-014-9534) [18], additional diffraction lines belonging to the set characterizing EuVO_4_ (PDF card no. 04-005-7366) were also present. The lack of diffraction lines characterizing orthorhombic V_2_O_5_ (PDF card no. 04-008-7123) indicates that during the synthesis of pentaeuropium decaoxovanadate (Eu_5_VO_10_), the reaction with the formation of EuVO_4_ takes place first in the substrate mixture at a temperature of about 600 °C according to the reaction:

Eu_2_O_3(s)_ + V_2_O_5(s)_ = 2 EuVO_4(s)_ (1)

The second stage of heating the sample, that is at 630 °C for 12 h, did not cause any changes in its phase composition, i.e., the sample above was two-phase and contained only europium(III) oxide and EuVO_4_. Compared to the first stage, the diffraction pattern of this sample differed only slightly in terms of the intensity of the recorded lines.

After the third stage of sample heating (1200 °C, 12 h), it was found that a reaction between europium(III) orthovanadate(V) and Eu_2_O_3_ had begun in the reaction mixture, forming a phase with X-ray characteristics not included in the PDF5+ database. However, based on the literature data [13] and the similarity of the recorded diffraction lines, i.e., their position and intensity, to those characterizing samarium(III) vanadate(V) with the formula Sm_5_VO_10_, it was found that the undescribed diffraction lines belong to the set of Eu_5_VO_10_ oxosalt. The synthesis reaction of pentaeuropium decaoxovanadate can therefore be represented by reaction Equation (2), which was confirmed by observing the change in the number of recorded reflections and their intensity after the next stage of sample heating.

EuVO_4(s)_ + 2 Eu_2_O_3(s)_ = Eu_5_VO_10(s)_ (2)

After the last heating step, i.e., the 1300 °C (12 h) sample was monophasic and contained only pentaeuropium decaoxovanadate. During the heating of the sample, its colour changed from an orange mixture of substrates, through the light grey colour of the mixture of EuVO_4_ with Eu_2_O_3_ until the light yellow of the compound Eu_5_VO_10_.

Due to the lack of information on the basic crystallographic data of the Eu_5_VO_10_ compound and a complete X-ray characterization of this phase in the available literature, the next stage of the work involved the indexing and refinement of the diffraction pattern of the europium(III) vanadate(V). The indexing and refinement were performed using the Expo2014 [19] and REFINEMENT software from DHN/PDS package, respectively. From the obtained solutions, the one with the highest figures of merit (FOM) was selected:

M_20_ = 6.4, F_20_ = 5.1, R_Q = 0.119%, mean|Δ2θ| = 0.031° Δρ = 0.50%

The results are presented in Table 1.

Based on the obtained indexing and refinement results, it was established that Eu_5_VO_10_ crystallizes in the monoclinic system, similarly to other lanthanide compounds with the general formula M_5_VO_10_ and the studied structure [13,20]. The calculated unit cell parameters are as follows:

- a = 8.975(5) Å;
- b = 7.959(4) Å;
- c = 13.834(9) Å;
- β = 92.73(5)°;
- V = 987.1(7) Å^3^;
- Z = 4;
- Space group: P2_1_ (No. 4).

The correctness of the presented results is indicated by a small difference between the density values calculated from the unit cell parameters (d_calc_ = 6.53 g/cm^3^) and determined experimentally using a gas pycnometer (d_exp_ = 6.50 g/cm^3^).

The next stage of the research was an attempt to obtain Eu_5_VO_10_ by methods alternative to the high-temperature one, i.e., mechanochemical and the modified Pechini method.

**Figure 3:**
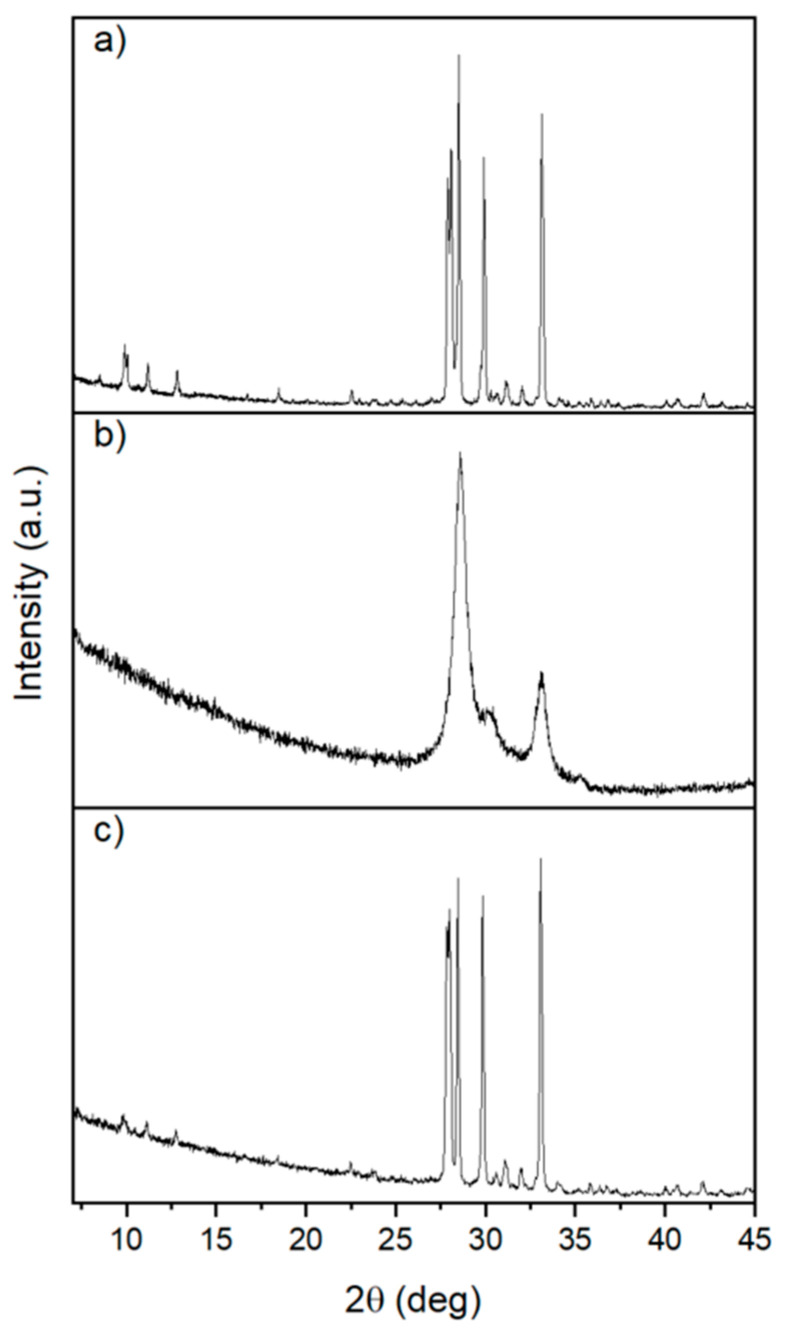
Fragments of diffractograms of the Eu_5_VO_10_ obtained by various methods: (**a**) ceramic, (**b**) mechanochemical, (**c**) modified Pechini method.

As a result of high-energy milling of the Eu_2_O_3_ and V_2_O_5_ mixture in a planetary ball mill, after ten hours of reaction, a product was obtained, whose registered diffraction pattern (Figure 3b) was similar to the diffraction pattern of Eu_5_VO_10_ synthesized by the ceramic method (Figure 3a).

The presented figure shows that the diffraction patterns of the europium(III) vanadate(V) obtained by these two methods differ in the number of recorded reflections with low relative intensity. This is primarily due to the fact that during the synthesis process in a high-energy planetary mill, multiple collisions of the grinding medium with the grounded material occur, which favours the crystallization of only selected lattice planes of the forming compound, with the highest intensity (I/I_0_) [21]. It cannot be ruled out that, only after grinding, the developed lattice planes are favoured in the crystallization process of the obtained europium(III) vanadate(V), as well as the fact that a certain portion of the product is in an amorphous form. It should also be noted that the significantly broadened reflections recorded in the Eu_5_VO_10_ diffraction pattern of the thus obtained compound indicate nanometric crystallite sizes, which was confirmed by Scherrer’s methods [22]. The calculated grain sizes of pentaeuropium decaoxovanadate are approximately 64 nm. For the calculations, the value of the shape factor a = 0.9 was assumed. After heating of the milled sample at 1300 °C during 3 h, registered XRD reflections were narrow and the number of them increased. The diffractogram of milled and heated Eu_5_VO_10_ looked similar to the patterns in Figure 3a,c. The diffraction pattern of the light yellow product obtained by the modified Pechini method (Figure 3c) was almost identical to that obtained by the ceramic method. Also in both cases, according to the Scherrer method, crystallites have around 0.15–0.20 μm size. Based on the observed diffraction line sets, it was determined that each of the three methods—ceramic, mechanochemical, and modified Pechini—could produce Eu_5_VO_10_ as the main reaction product.

### 3.2. SEM–EDX

**Figure 4:**
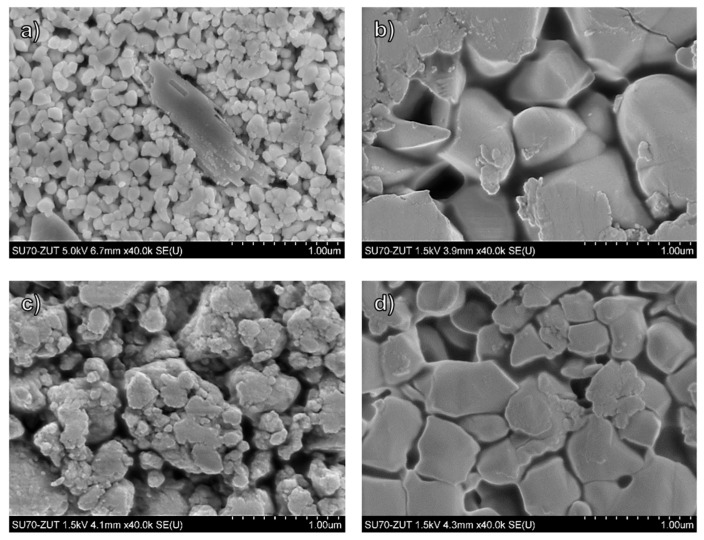
The SEM images of the samples contained: (**a**) the mixture of substrates after homogenisation by grinding; Eu_5_VO_10_ obtained by various methods: (**b**) ceramic, (**c**) mechanochemical, (**d**) modified Pechini method.

To confirm the size and shape of Eu_5_VO_10_ crystallites obtained by different methods, images were obtained using the SEM method, which are shown in Figure 4.

Based on the SEM images, it was found that Eu_5_VO_10_ crystallites have the shape of irregular polyhedra, and their sizes do not differ significantly between the high-temperature and modified Pechini synthesis methods. Their average sizes range from ~0.5 to 0.8 μm. In the case of synthesis carried out by the mechanochemical method, the crystallite sizes are varied and range from ~0.1 to ~0.4 μm. These values are in accordance with the calculated size of crystallites which confirms the fact that high-energy ball milling synthesis method promotes the formation of phases with much smaller crystallites, often of nano-sized sizes.

Moreover, to confirm the chemical composition of the obtained europium(III) vanadate(V), EDX analysis was conducted. The results of this analysis, that is average values from three chosen points of every sample, are presented in Table 2.

According to the obtained values of the atomic concentration of metallic elements in the Eu_5_VO_10_ compound it was observed that in every sample there are only small differences between the experimental and theoretical values. It confirms that the used chemical formula of the investigated europium(III) vanadate(V) is correct.

### 3.3. Thermal Stability Test

Due to the fact that no clear effects were registered on the DTA–TG curve of the obtained Eu_5_VO_10_ compound up to 1400 °C, in order to determine its thermal stability, the synthesized compound was additionally heated in a furnace in the temperature range of 1300–1400 °C. The furnace temperature was increased every 10 °C and the sample was thermostated at this temperature for 1 h.

After each heating step, the sample was homogenized and its phase composition was determined by XRD. Under the experimental conditions, a change in the number, position, and intensity of the recorded reflections was first observed after heating Eu_5_VO_10_ at 1310 °C. Heating the Eu_5_VO_10_ compound at this temperature resulted in its thermal decomposition, without any doubt, to Eu_2_O_3_ with a monoclinic structure (PDF card no. 04-004-2793) and a phase of as yet experimentally unconfirmed composition. At this stage of the research, it is very probable that, analogously to other compounds with the same molecular formula, e.g., Y_5_VO_10_ and Sm_5_VO_10_ [13], Eu_5_VO_10_ decomposes in the solid state according to the reaction equation:

2 Eu_5_VO_10(s)_ = Eu_2_O_3(s)_ + Eu_8_V_2_O_17(s)_ (3)

The solid state decomposition of the obtained compound is indicated by the fact that after heating Eu_5_VO_10_ at 1310 °C, it did not melt or even partially melt, which means that its decomposition products are characterized by higher melting and/or decomposition temperatures [23].

### 3.4. FTIR

**Figure 5:**
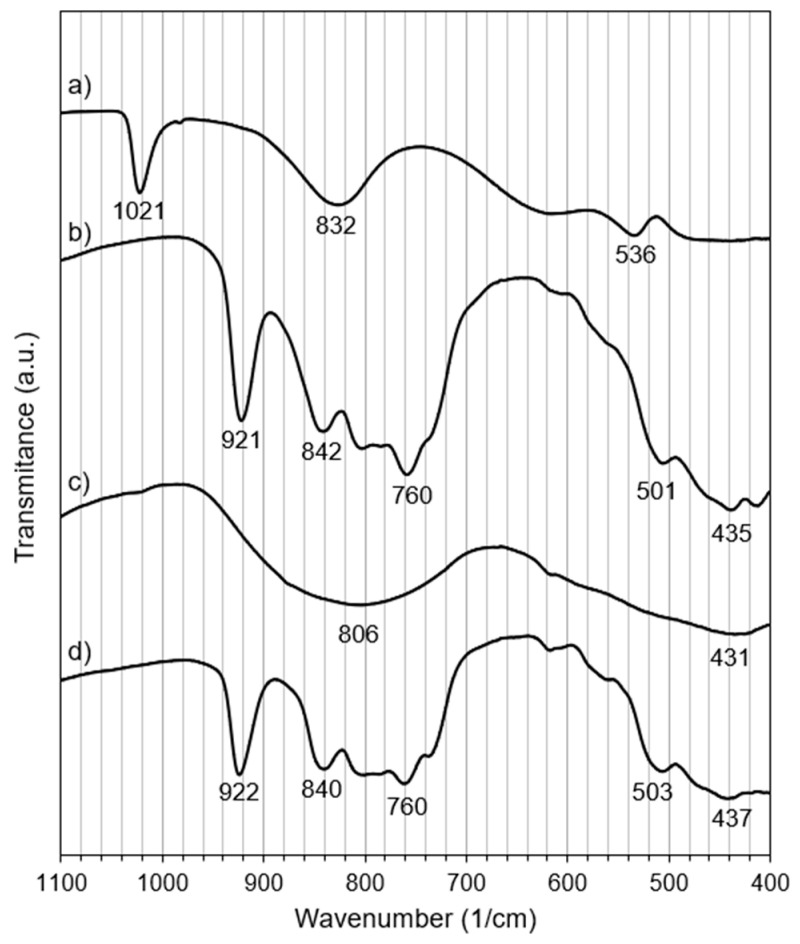
Fragments of IR spectra of the samples contained: (**a**) a mixture of the substrates; Eu_5_VO_10_ obtained by various methods: (**b**) ceramic, (**c**) mechanochemical, (**d**) modified Pechini method.

To determine the oxygen polyhedral that built the structure of Eu_5_VO_10_, FTIR studies were conducted. Comparative analysis of the spectra of the substrates and the resulting compound led to the conclusion that the IR spectrum of Eu_5_VO_10_ vanadate differs significantly from that of the substrate mixture, taking into account both the position of the absorption bands and their intensity. A summary of the recorded transmission spectra in the 400–1100 cm^−1^ range is presented in Figure 5.

The registered IR spectrum (Figure 5a) of the substrate mixture shows absorption bands which, according to the literature data, correspond to the stretching vibrations of the V–O [11] and Eu–O [24] bonds in the oxygen polyhedra of vanadium and europium. The absorption bands in the range of 700–950 cm^−1^, visible in the spectra in Figure 5b,d, according to the literature data, can be assigned both to the V–O–V bridge vibrations in the VO_4_ [11] polyhedra and to the Eu–O bonds in the EuO_8_ [24] polyhedra. Based on the similarity of the IR spectra of the obtained europium(III) vanadate(V) to other phases with the same formula type, it was concluded that, similarly to Sm_5_VO_10_ [13], the structure of Eu_5_VO_10_ is likely composed of VO_4_ tetrahedra [11] and EuO_8_ dodecahedra [24] connected by edges. It cannot be ruled out that the structure of the studied europium(III) vanadate(V) also includes EuO_6_ polyhedra [24]. It should be noted that in the case of the phase obtained by the mechanochemical method, one broad, diffuse absorption band (700–970 cm^−1^) was registered, which further confirms the presence of an amorphous phase in addition to the crystalline phase in the Eu_5_VO_10_ sample.

### 3.5. UV–VIS–DRS

The final step in this study was to estimate the energy gap of Eu_5_VO_10_ obtained by three different methods. To this end, a Tauc plot was created from the transformed UV–VIS spectrum, and then a tangent was drawn to the longest straight line segment.

**Figure 6:**
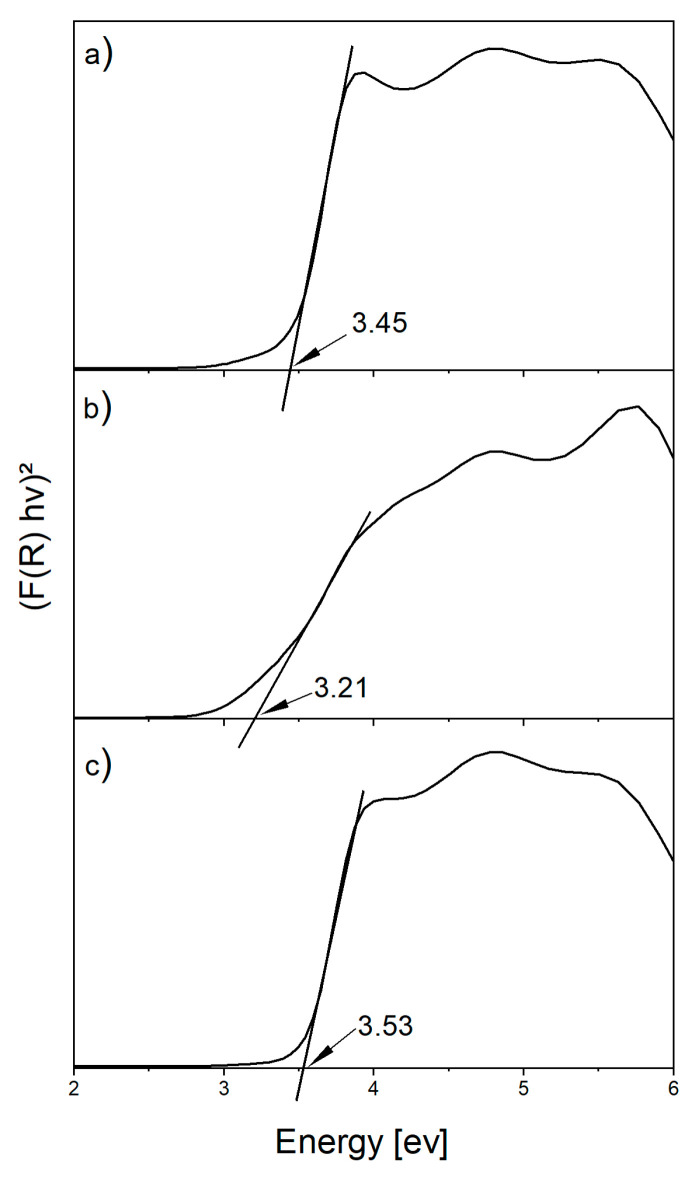
Summary of UV–VIS spectra after Tauc transformation: Eu_5_VO_10_ obtained by various methods: (**a**) ceramic, (**b**) mechanochemical, (**c**) modified Pechini method.

Figure 6 shows the UV–VIS spectrum after Tauc transformation of the Eu_5_VO_10_ compound obtained by both the ceramic and mechanochemical methods, as well as the modified Pechini method.

The intersection of the tangent line with the OX axis allows us to estimate the energy gap value. Based on the estimated E_g_ values, it was determined that, regardless of the synthesis method, Eu_5_VO_10_ belongs to the group of electrical semiconductors with a gap above 3.2 eV. Based on the literature data [25] concerning, among others, materials with a similar E_g_ value (ZnO, GaN, TiO_2_), it can be indicated that the obtained europium(III) vanadate(V) may find application as a component of phosphors, crystalline lasers, photocatalysts, etc.

## 4. Conclusions

The results of the conducted and presented studies showed, that:

- The Eu_5_VO_10_ compound formed in the binary V_2_O_5_–Eu_2_O_3_ oxide system can be synthesized both by high-temperature solid-state reactions and by the modified Pechini method to obtain a microcrystalline material, as well as by the mechanochemical method to obtain a nanocrystalline material with the participation of a microcrystalline material.
- The synthesis of Eu_5_VO_10_ occurs via an intermediate step, in which EuVO_4_ forms.
- Eu_5_VO_10_ crystallizes in the monoclinic system, with the following calculated unit cell parameters: a = 8.975(5) Å; b = 7.959(4) Å; c = 13.834(9) Å; β = 92.73(5)°. The number of molecules in the unit cell Z is four.
- The structure of the Eu_5_VO_10_ compound mainly consist of VO_4_ tetrahedrons and EuO_8_ dodecahedrons or EuO_6_ octahedrons.
- The Eu_5_VO_10_ compound is thermally stable in the air atmosphere up to the temperature around 1310 °C, above which it decomposes in the solid state to Eu_2_O_3_ and Eu_8_V_2_O_17_.
- Depending on the synthesis method used, the energy gap values are approximately around 3.45 eV for Eu_5_VO_10_ synthesized by the classical ceramic method, around 3.21 eV for the phase obtained by the mechanochemical method and around 3.53 eV for vanadate synthesized by the modified Pechini method.
- The band gap values indicate that regardless of the synthesis method, the Eu_5_VO_10_ compound is classified in the wide band gap electrical semiconductors group.

## Figures and Tables

**Table 1:** The results of the indexing of the Eu_5_VO_10_ powder diffractogram.

No.	hkl	d_obs_ [Å]	d_calc_ [Å]	I/I_0_ [%]
1	1 0 0	8.9740	8.9649	17
2	0 1 0	7.9338	7.9589	12
3	0 0 −2	6.9205	6.9094	12
4	−1 2 −2	3.2038	3.1932	71
5	1 0 4	3.1810	3.1734	78
6	2 0 3	3.1358	3.1387	100
7	0 2 −3	3.0056	3.0113	20
8	3 0 0	2.9904	2.9883	80
9	−1 2 3	2.8746	2.8818	9
10	−2 2 −2	2.7063	2.7023	91
11	4 0 3	1.9773	1.9786	17
12	−4 2 1	1.9456	1.9448	19
13	−4 1 −3	1.9192	1.9202	14
14	−1 3 5	1.8846	1.8844	15
15	−1 4 2	1.8746	1.8750	20
16	−1 1 −7	1.8558	1.8563	23
17	−4 0 6	1.6461	1.6459	11
18	−3 3 5	1.6369	1.6364	15
19	−4 2 5	1.6266	1.6270	11
20	0 5 0	1.5918	1.5918	12

**Table 2:** The results of the EDX analysis.

Element	Atomic Percent of Elements in Eu_5_VO_10_ Obtained with Methods:			Theoretical Values [% at.]
	High-Temperature	Mechanochemical	Modified Pechini	
Eu	84.2	84.0	84.8	83.3
V	15.8	16.0	15.2	16.7

## Data Availability

The original contributions presented in this study are included in the article. Further inquiries can be directed to the corresponding authors.
